# Shedding light on the next pandemic path, from outpatient to ICU, the effect of vitamin D deficiency in the SARS-CoV-2 pandemic

**DOI:** 10.3389/fnut.2023.1268267

**Published:** 2024-01-08

**Authors:** F. Celikmen, V. Tunaligil, E. C. Keles, D. S. M. Celikmen, S. Sarikaya

**Affiliations:** ^1^Department of Emergency Medicine, Yeditepe Medical School, Yeditepe University, Istanbul, Türkiye; ^2^Presidency of Disaster Health and Emergency Medical Services, TR MoH Health Directorate of Istanbul, Istanbul, Türkiye; ^3^Department of Biostatistics and Medical Informatics, Yeditepe Medical School, Yeditepe University, Istanbul, Türkiye; ^4^Department of Internal Medicine, Medical Park Goztepe Hospital, Bahcesehir University, Istanbul, Türkiye

**Keywords:** public health, emergency, disease outbreak, infection, international, prevention, syndemics, future healthcare systems

## Abstract

**Background:**

Vitamin D insufficiency is named “the pandemic of our era” by some experts. World Health Organization warns against a “deadlier outbreak” than the COVID-19 pandemic. Critical evidence is hereby for future pandemic prevention, with special emphasis on Vitamin D.

**Methods:**

A cross-sectional study was conducted with 172 unvaccinated adult participants, who presented to the emergency department. Blood measurements, radiological findings, and demographic features were evaluated in the four categories of “healthy adults, COVID-19 outpatients, hospitalized inpatients on the wards, and in the ICU.”

**Results:**

Results were statistically significant in association with age, gender, weight, Vitamin D, glucose, urea, creatinine, leucocyte, aspartate transaminase, hemoglobin, C-reactive protein, troponin, platelet/thrombocyte, ferritin, D-dimer, triglycerate, glycated haemoglobin, lactate dehydrogenase measurements, and chest computed tomography features (each *p* < 0.050).

**Conclusion:**

This article presents evidence to support the importance of Vitamin D for global public health. Patients with adequate levels of Vitamin D, glucose, urea, creatinine, leucocyte, aspartate transaminase, hemoglobin, C-reactive protein, troponin, platelet/thrombocyte, ferritin, D-dimer, triglycerate, glycated haemoglobin, lactate dehydrogenase are less likely to be admitted to ICU versus being outpatients. Factors include gender, age, weight, comorbidities, and computed tomography findings. The ultimate goal is to globally minimize preventable burdens of disease and death.

## Background and aims

Vitamin D insufficiency is a global health issue that afflicts more than one billion worldwide, named by some experts “the pandemic of our era” (1–3).

On World Health Organization (WHO) Director-General’s May 22, 2023 speech at the World Health Assembly (WHA), a critical present-day warning was is made about a “deadlier outbreak,” at a point when the number of confirmed cases had already reached nearly 767 million. Nearly seven million had lost their lives from confirmed severe acute respiratory syndrome coronavirus 2 (SARS-CoV-2). In the same time frame, WHO announces hundreds of new cases each week, shortly after an end to coronavirus disease 2019 (COVID-19) as a public health emergency was officially declared “with great hope” on May 4th, 2023 (4). The COVID-19 pandemic was declared a “public health emergency of international concern” on January 30th of 2020 by WHO Director-General and that it was “here to stay.” The global disease outbreak threatened lives and economies, having placed enormous and growing burdens on world populations. The virus and its varients continue to pose major challenges to scientists and clinicians, destined to exploring innovative ways to mitigate severe forms of the disease (5–8). Numerous articles calculted the potential risks of overlaps of COVID-19 with other universally common pathogens, such as the seasonal-influenza (8). “Last week, COVID-19 claimed a life every three minutes and that’s just the deaths we know about,” said WHO Director-General in May (4). It is time to advance and chart a clear path towards future pandemic prevention.

It is time to advance and chart a clear path towards future pandemic prevention. Vitamin D emerges with benefits in the fight against SARS-CoV-2 infection, yet there is need need for further analysis. Across the board, practice-based clinical judgement supports the notion that Vitamin D reduces COVID-19 severity. Logical line of reasoning underpins the proposition. Scientists around the world are committed to improving supporting evidence (9). Recent data have suggested a protective role of Vitamin D in COVID-19-related health outcomes since Vitamin D is known to play a crucial role in immune function and inflammation (6). Studies reported that patients with moderate–severe Vitamin D deficiency (<20 ng/mL) have a higher risk for in-hospital mortality due to COVID-19 than those with higher levels of Vitamin D (10–13). A growing body of literature raises the issue of Vitamins C and D for risk assessment and therapeutic options in COVID-19 (5). Various aspects of the relationship between Vitamin D status and COVID-19-related clinical outcomes remains controversial. There are several relatively small, single-site homogeneous populations studies based on assessment of a mainstream population for reference but with certain limits of generalizability (14). Systematic reviews underline that further research is urgently needed (15).

Vitamin D_3_ (cholecalciferol) is synthesized in skin by exposure to direct sunlight. Vitamin D_3_ is metabolized by the liver to 25-hydroxyvitamin D (calcidiol), then converted by the kidneys to 1,25-dihydroxyvitamin D (active form, calcitriol) (16). This article refers to 25-hydroxyvitamin D as Vitamin D, hence the best way to diagnose Vitamin D deficiency.

In large numbers, Vitamin D has been found to be low in COVID-19 patients and have been related to worse outcomes in several studies (17). In one investigation, Vitamin D levels were shown to predict the outcomes in 73 patients the clinical course of both 36 severe and 37 non-severe COVID-19 patients at hospitalization, when compared with 30 control subjects. Vitamin D levels at hospital-admission strongly predicted the occurrence of worsening outcomes in COVID-19, independent of the disease severity at presentation (18). Vitamin D deficiency was associated with a higher risk of COVID-19 hospitalization in a retrospective case–control study in England, which included 80,670 participants. Odds ratios for hospital admission were 2.3–2.4 times higher for levels <50 nmol/L, but without excess mortality risk. The conclusion was reached that widespread measurements and treatment may reduce the risk (19). A total of 288 COVID-19 patients participated in another research. Patients with lower albumin (*p* < 0.0005), lower Vitamin D (*p* = 0.002), higher D-dimer (*p* < 0.0005) levels had fatal disease outcomes had. Radiographic scores were increased in patients with lower serum albumin (*p* < 0.0005) and higher D-dimer (*p* < 0.0005) levels, but showed no statistically significant differences regarding Vitamin D concentrations (*p* = 0.261). An important combined role of “low Vitamin D, low albumin, high D-dimer” was pointed out in early diagnosis of severe disease as timely indicators (7). A single-center prospective study in India with 200 COVID-19 patients found no statistically significant differences in the length of hospital stay (*p* = 0.176), need for mechanical ventilation, or mortality, between normal and Vitamin D deficiencient levels <30 ng/mL (14).

A meta-analysis from five RCTs and trial sequential analysis presented definitive evidence on the protective effect of Vitamin D supplementation on COVID-19-related intensive care unit (ICU) and mortality. Vitamin D administration resulted in a decreased risk of death and ICU admission. The pooling of the studies was claimed to reach a definite sample size. The asessment was made that the association is conclusive (6). Epigenetic changes in COVID-19 patients were discussed in an early article which aimed to explore various processes contributing to disease severity (20). Evidence remains controversial. An Mendelian randomization study reported that genetically lowered serum Vitamin D concentrations are not causally associated with COVID-19 susceptibility, severity, or hospitalized traits, but did not evaluate the role of Vitamin D supplementation (21). Supplements may offer a relatively easy option to decrease the impact of the pandemic (9, 22). Strong evidence was presented on the benefits. Vitamin D deficiency of <20 ng/mL was identified in 14.5% of the 4,599 veterans with a positive SARS-CoV-2 test. A covariate-adjusted correlation was reported for 24.1–18.7% hospitalization (*p* = 0.009) and 10.4–5.7% mortality (*p* = 0.001) in the association of Vitamin D with COVID-19. Acknowledging that the sample of veterans disproportionately male, older, and with multiple comorbidities, multivariable analyses were conducted in terms of the generalizability of the findings, to adjust for the confounding factors, and still an independent effect of lower Vitamin D levels was found to be associated with COVID-related hospitalization and mortality. The study concluded that randomized controlled trials (RCTs) are needed to evaluate the impact (23). High-quality randomized controlled trials with larger populations are necessary to explore and define the role of Vitamin D supplementation in the prevention and treatment of COVID-19 (13, 21, 23–25).

A pilot study found low serum levels of Vitamins C and D in most of the cases in a cohort of 21 critically ill COVID-19 patients in ICU. Older age and low Vitamin C level appeared co-dependent risk factors for mortality (5). An observational, single-center, pilot study of limited sample size demonstrated that Vitamin D deficiency correlated with a reduced number of natural killer cells in 29 ICU and 10 non-ICU patients with COVID-19 pneumonia, with the evaluation that the beneficial effects of Vitamin D on protective immunity in the early stages were due in part to its effects on the innate immune system (26). The results of a single-center retrospective observational study, conducted with 40 ICU-admitted confirmed COVID-19 patients, indicated that serum Vitamin D ≤ 9.9 ng/mL on admission is a predictor of in-hospital mortality (10). A total of 83 confirmed COVID-19 cases were enrolled in a retrospective, observational, single-center respiratory-ICU study in Italy. The study demonstrated that moderate–severe Vitamin D hypoVitaminosis may predict worse prognosis and increased in-hospital mortality in patients with severe COVID-19 and acute respiratory failure (13). Decreased serum levels of Vitamin D versus the healthy participants was exhibited in a study with 100 participants, consisting of 50 healthy and 50 ICU-admitted COVID-19 (*p* = 0.0024). Findings suggested a probable association of Vitamin D concentrations with immunity and the risks (27). A study, which included 175 ICU-admitted COVID-19 patients with Vitamin D deficiency <12 ng/mL, aimed to investigate the relationship between clinical course and inhospital mortality. The study group (*n* = 113) received a high dose of 300,000 IU Vitamin D_3_ intramuscularly within the first 24 h of ICU admission, versus the control group (*n* = 62). Parenteral Vitamin D_3_ administration did not reduce the need for intubation, length of hospital stay, and inhospital mortality (17). A cross-sectional study included 194 adults with ICU-admitted COVID-19 patients, with results that confirmed the high prevalence of Vitamin D deficiency in severe cases (60.8%) and the positive association between Vitamin D deficiency, poor prognosis, and mortality due to secondary infections (28). Even stronger evidence was presented by a systematic review and meta-analysis on 2,078 patients from nine studies on the beneficial role of Vitamin D supplementation on ICU admission (*p* = 0.005), but not on mortality (*p* = 0.109) (15).

Resilience strategies for pandemic preparedness and evidence-based public health policy will enhance human well-being accross the populations. The end of a global health emergency does not mean the global health threat is over. A high-level leaders’ meeting will be held on pandemic preparedness and response at the annual WHA, set to address future pandemics. The commitment to a pandemic accord is important. The abiding threat of an “emerging deadlier pathogen” is a call for action. It is time to advance on future pandemic prevention and to chart a clear path forward towards that future (4). Mounting concern is now being voiced as the discoveries of global virology indicate that the natural order of the increasingly frequent pandemics in the coming future is inevitable (29).

In the pursuit to contribute to global health, the current cross-sectional research focuses on the role of Vitamin D levels in COVID-19 in improving clinical outcomes. The non-drug, single-center study was carried out under the coordination of Yeditepe University training hospital in Istanbul. In the metropolitan city, in a period of just over two-and-a-half months alone in 2020, ambulances responded to 35,403 SARS-CoV-2 emergency cases. The number of patients transported to-and-from state, private, and university hospitals were 29,762, 4,969, and 672 in consecutive order (30). Emergency ambulance services personnel had to design critical adaptive strategies in the complex environment of health care provision (31). The current study reports experiences from cases which originated in the emergency department of the university hospital and were either discharged for at-home follow-up or referred for hospitalization on the wards or in the ICU of the same health care institution. Demographic information of gender, age, weight, height, blood concentrations of Vitamin D, glucose, urea, creatinine, leucocyte, aspartate transaminase (AST), alanine transaminase (ALT), hemoglobin (Hgb), C-reactive protein (CRP), troponin, platelet/thrombocyte, ferritin, D-dimer, triglycerate, glycated haemoglobin (HbA1c), and lactate dehydrogenase (LDH), fibrinogen, and creatine kinase (CK), and chest CT testing were evaluated. Main emphasis was given to investigating the effect of Vitamin D levels on patient prognosis in COVID-19 patients. The study aims to shed light on the next pandemic path and to contribute to clinical competencies and public health policies for mass protection againt brutal killers of the future. The ultimate goal is to minimize the preventable impacts of morbidity and mortality on world populations.

## Materials and methods

### Ethics committee approval

Based on the application with file #1866, the Clinical Research Ethics Committee at Yeditepe University in Istanbul gave their approval on May 6 of 2020, with decision #1203.

The records that might reveal the identity of the volunteers were kept confidential by giving a “volunteer code” which cannot be disclosed to the public. The identity of the volunteer was assured to remain confidential even if the research results are published. By signing the written informed consent form, where viewers, polling persons, ethics committee, institution and other relevant health authorities may have direct access to your original medical records, but this information will be kept confidential, the volunteer or legal representative will have authorized such access. No payment or extra services were offered to the volunteers.

### Research design

A cross-sectional research study was conducted. The aim was to investigate the effect of Vitamin D levels on patient prognosis in COVID-19 patients, shedding light on the next pandemic. The non-drug, single-center study was carried out under the coordination of Yeditepe University Faculty of Medicine Department of Emergency Medicine at the university-affiliated Kozyatagi Hospital in Istanbul. Data was collected in a period of 3 months, June 1–September 1, 2020. Blood Vitamin D, glucose, urea, creatinine, leucocyte, aspartate transaminase (AST), alanine transaminase (ALT), fibrinogen, creatine kinase (CK), hemoglobin (Hgb), C-reactive protein (CRP), troponin, platelet/thrombocyte, ferritin, D-dimer, triglycerate, glycated haemoglobin (HbA1c), lactate dehydrogenase (LDH) levels were measured. Demographic information, such as gender, age, weight, height was collected for the total number of 172 participants, adults aged ≥18, not vaccinated against COVID-19, who presented to the emergency department of the hospital. A “COVID-19 Patient Follow-up Form” was used in the evaluation. With voluntary participation and comprehension of informed consent, healthy adults and COVID-19 patients who met the Turkish Ministry of Health (TR MoH)‘s case definition criteria were included in the study. In accordance with updated TR MoH algorithm and guidelines, confirmed cases of COVID-19 were diagnosed by positive Polymerase Chain Reaction (PCR) testing before or during ward/ICU hospitalization. The distribution of the participants was 43 healthy adults, 43 COVID-19 outpatients, 43 COVID-19 hospitalized inpatients on the wards, 43 COVID-19 hospitalized patients in the ICU. Blood measurements and demographic features were compared between the four groups. Statistically significant results of clinical importance are reported in this paper. Probable cases of COVID-19 with negative PCR results were excluded from the study. The conduct of research posed no risks for the volunteers, who were not subjected to any procedures other than blood testing used for scientific research.

### Statistical analysis

The data were analyzed using the Statistical Packages of Social Sciences (SPSS) 28.0 program on the computer. Descriptive statistics were presented as mean ± standard deviation, median, minimum and maximum values for continuous variables, and as frequency and percentages for categorical variables. The conformity of the data to the normal distribution was evaluated with the Kolmogorov Smirnov test. Two independent samples *t*-test was used to compare the means of the measurements with normal distribution between the groups, and the Mann–Whitney U test was used to compare the measurement values that did not fit the normal distribution. Chi-square test or Fisher exact probability test was used to compare categorical variables. If *p* < 0.05, the difference was considered significant.

### The clinical characteristics of the patients at diagnosis

TR MoH diagnostic guidelines, treatment algorithms, and updates for COVID-19 were followed in patient management. Patients were grouped under three categories, as COVID-19 outpatients, hospitalized inpatients on the wards, and in the ICU. Outpatients were followed in home isolation, without hospitalization, with treatment recommendations were < 50 years of age, without comorbidities, with normal imaging and mild pneumonia, blood lymphocyte counts ≥800/μL, serum CRP ≤ 40 mg/L, ferritin ≤500 ng/mL, D-dimer ≤ 1,000 ng/mL. Patients with findings which indicated severe course of the disease were hospitalized and monitored. These included mild-to-moderate pneumonia with respiratory rates ≥24/min, fingertip oxygen saturation (SpO2) ≤93%, mild-to-moderate pneumonia with poor prognostic criteria in the blood tests at admission, such as blood lymphocyte count <800/μL or serum CRP > 10 mg/L × upper limit of normal value or ferritin >500 ng/mL or D-dimer > 1,000 ng/mL, and severe pneumonia with changes in consciousness, respiratory distress, tachypnea or tachycardia with respiratory rates ≥30 min, SpO2 ≤ 90%, bilateral widespread >50% involvement in lung imaging, hypotension <90/60 mmHg, mean blood pressure < 65 mmHg, tachycardia >100, sepsis, septic shock, myocarditis, acute coronary syndrome, arrhythmia, acute kidney injury. Hospitalization was indicated in additional situations deemed necessary in by the consulting physician. Indications for ICU admission were respiratory rate > 30/min, signs of dyspnea and respiratory distress, oxygen saturation < 90% despite nasal oxygen support ≥5 L/min, partial pressure of oxygen in arterial blood (PaO2) <70 mmHg despite nasal oxygen support ≥5 L/min, partial pressure of oxygen in arterial blood to the fraction of inspiratory oxygen concentration (PaO2/FiO2) <300, lactate >4 mmol/L, bilateral infiltrates or multi-lobar involvement on chest radiography or tomography, hypotension with systolic blood pressure (SBP) < 90 mmHg, >40 mmHg decrease from regular SBP, mean arterial pressure < 65 mmHg, poor skin perfusion, organ dysfunction such as kidney function test, liver function test disorder, thrombocytopenia, confusion/disorientation, in the presence of immunosuppressive disease, underlying comorbidities, uncontrolled medical conditions, troponin elevation, arrhythmia. Admission to ICU critical care was indicated in additional situations deemed necessary in by the consulting physician.

Discharge criteria were the absence of fever, the need for oxygen for at least 48–72 h, and the clinician’s approval. Antiviral agents targeting the virus were administered according to the TR MoH treatment guidelines for adults. The patient approach included additional clinical considerations for each individual, along with the therapeutics. Needs were assessed. Monitoring of symptoms, comorbities, and the optimal management of nutritional deficiencies were carried out, when adequate. Drug dosages, routes of administration, and the duration of treatment were outlined in the manuals for the three patient categories. As recommended, *favipiravir and/or hydroxychloroquine* were used for confirmed asymptomatic COVID-19 cases and those with uncomplicated mild-to-moderate pneumonia, who were also followed as outpatients. The same medications were used with different regimens for hospitalized probable/confirmed COVID-19 cases, including those who were uncomplicated or patients with mild-to-moderate and severe pneumonia. A large-scale study reported from Istanbul statistically significant differences in ICU admissions from 24% to 12%, and in intubations from 77% to 66%, following the addition of *favipiravir* to the national COVID-19 treatment protocol (32).

## Results

**Figure 1:**
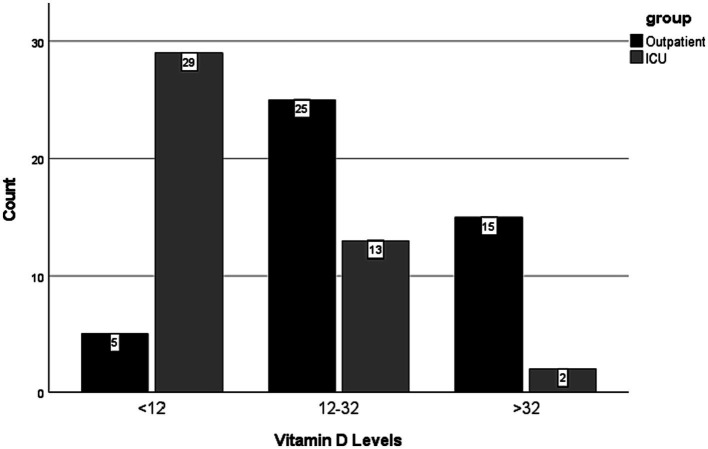
Vitamin D levels by the groups, patients in intensive care units (ICU) versus the outpatients.

Results were statistically significant in association with age (*p <* 0.001), weight (*p =* 0.001), Vitamin D (*p <* 0.001), glucose (*p <* 0.001), urea (*p <* 0.001), creatinine (*p =* 0.005), leucocyte (*p <* 0.001), aspartate transaminase (AST) (*p <* 0.001), hemoglobin (Hgb) (*p <* 0.001), C-reactive protein (CRP) (*p <* 0.001), troponin (*p <* 0.001), platelet/thrombocyte (*p =* 0.015), ferritin (*p =* 0.004), D-dimer (*p <* 0.001), triglycerate (*p =* 0.019), glycated haemoglobin (HbA1c) (*p =* 0.014), and lactate dehydrogenase (LDH) (*p <* 0.001). Vitamin D and Hgb levels were lower in ICU patients, compared to outpatients (Figure 1). All other measurements were higher in ICU patients, compared to outpatients. No statistically significant results were found to be associated with height (*p =* 0.565), levels of alanine transaminase (ALT) (*p =* 0.389), fibrinogen (*p =* 0.103), and creatine kinase (CK) (*p =* 0.193) (Table 1).

**Table 1:** Comparisons of demographic factors and blood measurements by the groups, patients in intensive care units (ICU) versus the outpatients.

	Group	Mean	*n*	Std. deviation	Median	Minimum	Maximum	*p* value
Age	ICU	66.6818	44	13.65994	65.5000	24.00	102.00	<0.001^a^*
	Outpatient	45.3778	45	13.16726	47.0000	22.00	77.00	
	Total	55.9101	89	17.10571	57.0000	22.00	102.00	
Weight	ICU	78.7500	44	7.85368	78.5000	63.00	96.00	0.001^a^*
	Outpatient	70.5556	45	14.54391	70.0000	50.00	108.00	
	Total	74.6067	89	12.36437	76.0000	50.00	108.00	
Height	ICU	169.5455	44	6.90956	169.0000	158.00	182.00	0.565^a^
	Outpatient	168.6222	45	8.09121	165.0000	158.00	188.00	
	Total	169.0787	89	7.50186	167.0000	158.00	188.00	
Vitamin D	ICU	11.5911	44	11.11248	8.2300	0.76	63.00	<0.001^b^*
	Outpatient	31.0987	45	17.66086	26.4800	5.33	91.93	
	Total	21.4545	89	17.67762	18.9200	0.76	91.93	
Glucose	ICU	175.3636	44	67.78698	156.0000	98.00	366.00	<0.001^b^*
	Outpatient	111.1852	27	20.18575	107.0000	87.00	166.00	
	Total	150.9577	71	62.91728	126.0000	87.00	366.00	
Urea	ICU	77.7955	44	47.57837	60.0000	15.00	213.00	<0.001^b^*
	Outpatient	11.5714	42	3.36512	11.5000	7.00	23.00	
	Total	45.4535	86	47.53244	22.0000	7.00	213.00	
Creatinine	ICU	1.4332	44	1.74797	0.9000	0.39	10.00	0.005^b^*
	Outpatient	0.7336	42	0.16894	0.7200	0.40	1.12	
	Total	1.0915	86	1.29737	0.7500	0.39	10.00	
Leucocyte	ICU	12251.5909	44	6709.85355	9900.0000	2100.00	33500.00	<0.001^b^*
	Outpatient	5947.7273	44	2144.92247	5850.0000	2300.00	11000.00	
	Total	9099.6591	88	5880.05873	7550.0000	2100.00	33500.00	
Aspartate transaminase (AST)	ICU	67.8864	44	91.58075	46.5000	10.00	616.00	<0.001^b^*
	Outpatient	35.4286	42	37.28738	23.0000	13.00	195.00	
	Total	52.0349	86	71.97098	34.5000	10.00	616.00	
Alanine transaminase (ALT)	ICU	43.3636	44	46.63370	27.5000	6.00	283.00	0.389^b^
	Outpatient	36.8095	42	35.06665	23.0000	8.00	174.00	
	Total	40.1628	86	41.28121	25.0000	6.00	283.00	
Fibrinogen	ICU	426.0682	44	180.03584	376.0000	173.00	971.00	0.103^b^
	Outpatient	507.3750	8	144.71838	466.5000	378.00	819.00	
	Total	438.5769	52	176.29680	405.5000	173.00	971.00	
Creatine kinase (CK)	ICU	469.6364	44	1095.43915	105.0000	0.00	5316.00	0.193^b^
	Outpatient	87.5000	4	91.59512	60.0000	11.00	219.00	
	Total	437.7917	48	1053.46484	99.5000	0.00	5316.00	
Hemoglobin (Hgb)	ICU	11.8977	44	2.39423	12.1500	6.90	18.40	<0.001^a^*
	Outpatient	13.4795	44	1.55662	13.6500	8.70	16.70	
	Total	12.6886	88	2.15952	12.9000	6.90	18.40	
C-reactive protein (CRP)	ICU	175.3682	44	125.54618	159.5000	6.70	513.00	<0.001^a^*
	Outpatient	18.3953	43	24.87602	8.0000	1.00	95.00	
	Total	97.7839	87	120.05856	34.0000	1.00	513.00	
Troponin	ICU	237.4136	44	616.96710	27.0000	0.40	2761.00	<0.001^b^*
	Outpatient	0.0105	6	0.00122	0.0100	0.01	0.01	
	Total	208.9253	50	583.19079	24.5000	0.01	2761.00	
Platelet/thrombocyte	ICU	291739.3864	44	144229.62025	282500.0000	229.00	877000.00	0.015^a^*
	Outpatient	231090.9091	44	72574.52246	226000.0000	65000.00	393000.00	
	Total	261415.1477	88	117536.92265	251000.0000	229.00	877000.00	
Ferritin	ICU	506.8818	44	422.85096	388.5000	13.00	1500.00	0.004^b^*
	Outpatient	490.0000	39	1530.15393	131.0000	13.00	9680.00	
	Total	498.9494	83	1085.75318	316.0000	13.00	9680.00	
D-dimer	ICU	1.6441	44	1.42420	1.3000	0.10	7.10	<0.001^b^*
	Outpatient	0.5019	32	0.42675	0.3700	0.04	2.08	
	Total	1.1632	76	1.24918	0.6000	0.04	7.10	
Triglycerate	ICU	186.2857	42	85.08655	181.5000	58.00	388.00	0.019^a^*
	Outpatient	128.2667	15	63.49631	111.0000	55.00	313.00	
	Total	171.0175	57	83.50353	153.0000	55.00	388.00	
Glycated haemoglobin (HbA1c)	ICU	6.6523	44	1.32533	6.3000	4.90	11.70	0.014^b^*
	Outpatient	5.7200	10	0.70522	5.5500	5.00	7.30	
	Total	6.4796	54	1.28186	6.2000	4.90	11.70	
Lactate dehydrogenase (LDH)	ICU	501.3182	44	212.23142	478.0000	129.00	1059.00	<0.001^a^*
	Outpatient	205.8387	31	106.73678	168.0000	124.00	640.00	
	Total	379.1867	75	228.58080	303.0000	124.00	1059.00	

*p < 0.050 statististically significant.

Reference ranges for blood tests: Vitamin D (<12 ng/dL deficient, 12–32 ng/dL insufficient, >32–150 adequate, 150 high, possible toxicity), glucose (<100 mg/dL), urea (<50 mg/dL), creatinine (0.5–0.9 mg/dL for women, 0.7–1.2 mg/dL for men), leucocyte (5,000–10,000/mm), AST (<35 units/L for women, <48 units/L for men), ALT (<35 units/L for women, <46 units/L for men), Fibrinogen (190–430 mg/dL), CK (26–192 U/L for women, 39–308 U/L for men), Hgb (11.7–16 g/dL for women, 13.1–17.2 g/dL for men), CRP (<5 mg/L), troponin (<0.16 ng/mL), thrombocyte (150,000–450,000 platelets/mcL), ferritin (10–350 ng/mL for women, 30–400 ng/mL for men), D-dimer (<0.42 mg/L), triglycerate (37–148 mg/dL), HbA1c (<4.5%), LDH (135–215 units/L for women, 135–225 units/L for men).

a Two independent samples *t*-test.

b Mann-Whitney U test.

**Figure 2:**
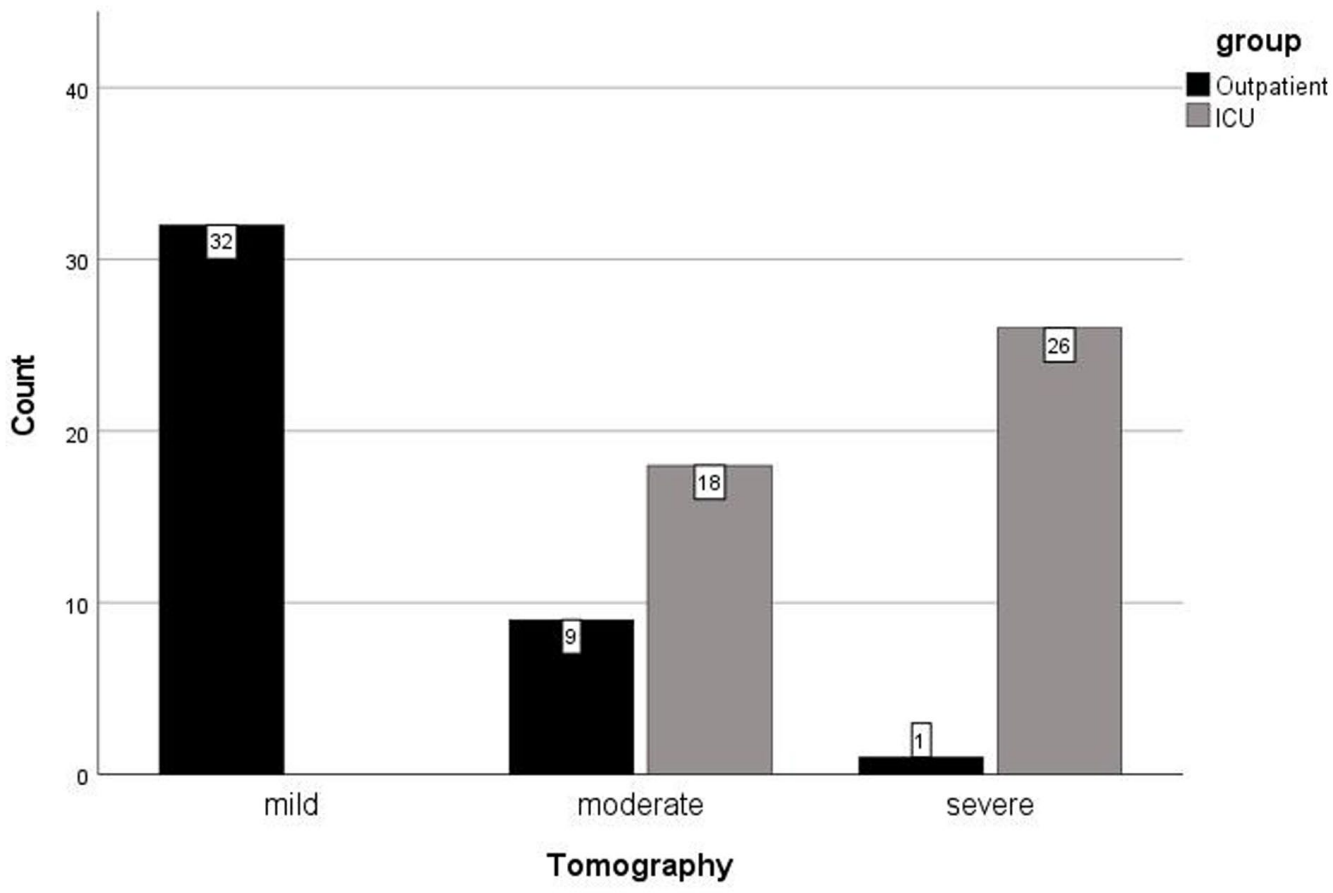
Computer tomography chest imaging manifestations by the groups, patients in intensive care units (ICU) versus the outpatients.

Results were statistically significant in association with gender (*p* = 0.020). There were more males than females in the ICU (29:15, 61.7:35.7%). Results were statistically significant in association with comorbidity (*p* < 0.001). There were more patients with comorbidities in the ICU than outpatients with comorbidities (39:20, 66.1:33.9%). Results were statistically significant in association with Vitamin D level (*p* < 0.001). There were more patients with severe Vitamin D deficiency in the ICU than outpatients with severe Vitamin D deficiency (29:5, 85.3:14.7%). Results were statistically significant in association with chest CT features (*p* < 0.001). All patients with mild chest CT features were outpatients (32, 100%) (Figure 2). Nearly all patients with severe chest CT findings were in the ICU, except for one outpatient with severe chest CT findings (26:1, 96.3:3.7%) (Table 2).

**Table 2:** Gender, comorbidity, Vitamin D levels, and computer tomography (CT) chest imaging comparisons by the groups, patients in intensive care units (ICU) versus the outpatients.

			Group		Total	*p* value
			ICU	Outpatient		
Gender comparisons by the groups						
Gender	Female	Count	15_a_	27_b_	42	0.020*
		% within gender	35.7%	64.3%	100.0%	
	Male	Count	29_a_	18_b_	47	
		% within gender	61.7%	38.3%	100.0%	
Total		Count	44	45	89	
		% within gender	49.4%	50.6%	100.0%	
Comorbidity comparisons by the groups						
Comorbidity	No	Count	5_a_	25_b_	30	<0.001*
		% within comorbidity	16.7%	83.3%	100.0%	
	Yes	Count	39_a_	20_b_	59	
		% within comorbidity	66.1%	33.9%	100.0%	
Total		Count	44	45	89	
		% within comorbidity	49.4%	50.6%	100.0%	
Vitamin D level comparisons by the groups						
Vitamin D	Severe deficiency	Count	29_a_	5_b_	34	<0.001*
		% within Vitamin D level	85.3%	14.7%	100.0%	
	Moderate deficiency	Count	13_a_	25_b_	38	
		% within Vitamin D level	34.2%	65.8%	100.0%	
	Normal	Count	2_a_	15_b_	17	
		% within Vitamin D level	11.8%	88.2%	100.0%	
Total		Count	44	45	89	
		% within Vitamin D level	49.4%	50.6%	100.0%	
CT chest imaging comparisons of by the groups						
CT	mild	Count	0_a_	32_b_	32	<0.001*
		% within CT	0.0%	100.0%	100.0%	
	moderate	Count	18_a_	9_a_	27	
		% within CT	66.7%	33.3%	100.0%	
	severe	Count	26_a_	1_b_	27	
		% within CT	96.3%	3.7%	100.0%	
Total		Count	44	42	86	
		% within CT	51.2%	48.8%	100.0%	

Chi-square test. *p < 0.050 statistically significant. Each subscript letter denotes a subset of group categories whose column proportions do not differ significantly from each other at the 0.05 level. Fisher’s Exact test. *p < 0.050 statistically significant. Each subscript letter denotes a subset of grup categories whose column proportions do not differ significantly from each other at the 0.05 level.

## Discussion

Blood measurements, radiological findings, and demographic features were compared between the four groups of the total number of 172 participants. Even numbers of healthy adults, COVID-19 outpatients, and inpatients hospitalized on the wards or the ICU were enrolled. Statistically significant results of clinical importance are reported in this paper, in terms of the critical implications for pandemic preparedness and public health.

Results were statistically significant in association with age, weight, Vitamin D, glucose, urea, creatinine, leucocyte, AST, Hgb, CRP, troponin, platelet/thrombocyte, ferritin, D-dimer, triglycerate, HbA1c, and LDH measurements (*p* < 0.050). Patients admitted to ICU were detected to have lower than normal Vitamin D and Hgb levels, compared to outpatients. All other measurements were higher in ICU patients, compared to outpatients. No statistically significant results associated with height, levels of alanine transaminase (ALT), fibrinogen, and creatine kinase (*p* > 0.05) (Table 1). Results were statistically significant in association with gender, comorbidity, Vitamin D levels, and chest CT findings (*p* < 0.050). In the outpatient setting, there were more females than males (64.3 versus 38.3%). When compared to outpatients, there were more males than females (61.7 versus 35.7%), more patients with comorbidities than those without (66.1 versus 33.9%), more patients with more severe levels of Vitamin D deficiency (85.3 versus 14.7%), and more patients with severe chest CT findings (96.3 versus 3.7%) in the ICU. Being male, having comorbidities, lower levels of Vitamin D, and severe versus mild chest CT findings might evidently be predisposing factors for admission to ICU for COVID-19 patients in the current study (Table 2).

Evidence from current research suggests particular focus on protection from Vitamin D, to reduce the risks and complications. Findings underscore the benefits of healthy Vitamin D levels. These evaluations are consistent with findings from existing literature. The study concluded that COVID-19 prognosis is poorer in patients with concurrent conditions, a recognizable inference. The presence of additional conditions was a factor associated with higher chances of being admitted to ICUs (6, 7, 10–13, 15, 17–19, 23, 28, 33). On the pressing issue of killer pandemics, researchers of the current research agree with previous studies, which indicated that further detailed analysis, stronger evidence with larger sample sizes, and valid research techniques are crucial to increase generalizability (10, 13, 15, 21, 23–25). A meaningful aspect of the study is also the fact that subjects included in the study had not been previously vaccinated for COVID-19. Since it is impossible to go back in time, there will never be a study group without vaccine-induced immunity or the same level of naturally-acquired active immunity in history again. The study interval is representative of an important period to better observe the effects of Vitamin D.

There are certain limitations to the study, which may altrenatively be develop into assets for future research. This cross-sectional study collected data to examine the associations between the blood measurements, radiological findings, demographic features in the four categories of being healthy adults, COVID-19 outpatients, hospitalized inpatients on the wards, and in the ICU. These evaluations generated valuable information, but there were certain limitations in terms of the outcome of the study. The research findings show that vitamin D deficiency may be a prognostic factor but cannot be conclusively attributed as the causative factor. This limitation arises from the cross-sectional nature of the study, in which baseline features were measured. The identified associations may or may not definitively correlate with survival outcomes. Due to the constraints in the study design, while the results reveal significant associations, a direct cause-and-effect relationship cannot be inferred between Vitamin D deficiency and ICU admission. This descriptive cross-sectional study characterized the prevalence of and the disease outcomes in the COVID-19 population under investigation. This prevalence study examined the data on COVID-19 and Vitamin D status at one particular time point. Results of the cross-sectional study helps generate the hypothesis that may shedding light on the next pandemic path and illuminate that a similar longitudinal study is worth the investment, lending to the creation of stronger cohort studies or randomized control trials. Causality should be further investigated. The results obtained in the study prove that large nutritional studies should be planned to show the clear benefits of vitamin D status for COVID-19 prognosis.

At the time the research was run, delimitations were set to reflect a broader picture in the management of COVID-19 patients. Specific decisions were made at the intiation of the study, at a time when vaccinations were not yet available and little was known worldwide about the disease. Since this study did not aim to investigate vitamin D metabolism or bone development, the carrier protein albumin levels were not taken into account. Preliminary results are promising. Research outcomes were quickly implemented into clinical practice because COVID-19 was a pressing issue with high levels of mortality, and there was a darth of definitive evidence on how to approach the patients. The world is at the next stage now, with accumulated knowledge from various sources. Thus, we are presently ready to take a more refined approach to obtain categorical evidence.

Study findings suggest that further assessments of nutritional deficiencies are worthwhile. The scope should be expanded to help measure nutritional value. Further research methodology should describe additional components, beyond the value of Vitamin D. Assessments should include the measurement of serum albumin. Several tests serve to evaluate nutritional status, including biochemical data, medical findings, procedures, nutrition-focused physical examination, analysis of the size, functional capacity, and history of the patient. Serum visceral proteins, such as albumin and prealbumin of shorter half-life, are used as markers of the nutritional status and prognosis predictors, especially in the presence of multiple-organ disease involvement. Easily measurable biomarkers of malnutrition, such as prealbumin, may be of interest, along with thorough physical examination, as predictors of the prognosis for surgical outcomes and of mortality in severe illnesses. Other markers of the nutritional status such as urinary creatinine or 3-methylhistidine as indicators of muscle protein breakdown have not found widespread use. Vitamin D deficiency is common and has detrimental effects on musculoskeletal health, cardiovascular disease, autoimmunity and cancer. Studies have suggested a possible interaction between vitamin D and IGF-1. The usefulness of serum insulin-like growth factor-1 concentration as a specific marker for detecting malnutrition is clinically controversial (34, 35). In future hospital-based cross-sectional studies, it is suggested to highlight the role of vitamin D by adding more relevant biomarkers or variables that can relate to the mechanism of action. Advanced investigations and reevaluations are critical, to know the true impact, as this information is also pertinent to group comorbidities.

The idea of vitamin D’s role in immune response is well-established. The protective effect of Vitamin D in preventing disease exacerbations, intensive care hospitalization, and mortality is widely recognized. In our study, it was observed that people with low vitamin D levels suffered from SARS Cov 2 more severely than those with high vitamin D levels. Current research presents novel evidence from Istanbul, on the prognostic and therapeutic role of Vitamin D in COVID-19. This study supports previous evidence from different locations. Considering the impact of geography on vitamin D status, the significance of the study is implied in its location. Studies need to be intensified in this region of the world, which offers rich dynamics regarding vitamin D deficiencies. Vitamin D deficiency remains an important problem in Turkey, despite the abundance of sunlight in the country (36, 37). In a study of prevalence over 60% of Turkish adults were found to be Vitamin D deficient (38). A meta-analysis, with a sample size of 111,582 from 40 studies, estimated the prevalence of Vitamin D deficiency to be 63% for the overall population (37). Lack of nutrition knowledge and poor dietary habits, excessive amount of time spent indoors, conservative dressing style covering a significant part of the skin may be some of the most commun causes. Scholarly views have been also expressed that this may be related to an enzyme defect or lack of intestinal absorption in the Turkish population (39). There are studies from two other cities, namely Izmir and Canakkale, in Turkey which investigated whether vitamin D levels are associated with the need for mechanical ventilation, ICU admission and length of stay, hospitalization, and COVID-19 related in-hospital mortality in critically ill COVID-19 patients (40, 41). It is thus important, to emphasize the importance of regional studies.

This article fulfills an important task in emphasizing that Vitamin D may potentially be an invaluable mitigation tool and prevent the next pandemic from turning into a global public health crisis (42).

## Conclusion

Is your blood cholecalciferol ready for the next pandemic? The effect of Vitamin D deficiency in the SARS-CoV-2 pandemic sheds light on the next pandemic path, perhaps for the future of humankind. Results of the current study show that measurable variables of health are meaningful prognostic predictors of COVID-19 severity. Even though conclusive evidence is not presented as proof of causality, vitamin D deficiency emerges as a prominent prognostic factor, in the cross-sectional research. Further investigation is suggested, based on the results of which indicate that patients with higher Vitamin D and Hgb blood measurements are seemingly less likely to be admitted to ICU and more likely to be treated as outpatients. Gender, age, comorbidities, chest CT findings, weight, glucose, urea, creatinine, leukocyte, AST, CRP, troponin, platelet/thrombocyte, ferritin, D-dimer, triglyceride, HbA1c, and LDH are all factors which impact disease severity and outcomes in predicted patterns.

This article highlights the importance of Vitamin D for global public health, by presenting evidence from Istanbul, a metropolis of two continents. Eminent factors affecting COVID-19 prognosis are reported in an effort to improve the outcomes of future outbreaks, with hard-earned lessons from recent universal experiences. There will inevitably be new pandemic agents, some more lethal than SARS-CoV-2, but we can reduce the risks.

The current topic remains an urgent matter of critical importance. The ultimate goal is to globally minimize preventable burdens of disease and death.

## Data availability statement

The raw data supporting the conclusions of this article will be made available by the authors, without undue reservation.

## Ethics statement

The studies involving humans were approved by based on the application with file #1866, the Clinical Research Ethics Committee at Yeditepe University in Istanbul gave their approval on May 6 of 2020, with decision #1203. The studies were conducted in accordance with the local legislation and institutional requirements. The participants provided their written informed consent to participate in this study.

## Author contributions

FC: Conceptualization, Data curation, Funding acquisition, Investigation, Methodology, Project administration, Resources, Supervision, Validation, Writing – review & editing. VT: Conceptualization, Investigation, Writing – original draft, Writing – review & editing, Methodology, Supervision. EK: Formal analysis, Methodology, Writing – review & editing. DC: Investigation, Validation, Writing – review & editing. SS: Supervision, Validation, Writing – review & editing.
